# Supplementary material for: Are carbon emissions trading and green financial instruments synergistic? -Comprehensive quantitative research based on content analysis
Source: PLoS One. 2024 Mar 7;19(3):e0298601. doi: 10.1371/journal.pone.0298601 (PMC10919601; doi:10.1371/journal.pone.0298601)
Supplement: S1 Data — (ZIP) [file pone.0298601.s001.zip › Data/地级市Z_F工作报告.zip!/░▓╗╒/│n╓▌╩╨/└┤╫╘╣1⁄2╓┌║┼í╛┬φ┐╦╩2╛▌═°í┐_2012.docx]

各位代表：

　　现在，我代表市人民政府，向大会作政府工作报告，请予审议，并请市政协委员和其他列席人员提出意见。

　　一、2011年工作回顾

　　2011年是我市迎难而上、破难而进的一年，也是我市努力拼搏、奋力赶超的一年。这一年，国际金融危机演变为欧美主权债务危机，国际形势异常严峻；这一年，国内经济运行矛盾叠加、困难重重，用钱用地既贵又难，用工用电既缺又限，节能减排既紧又严，市场竞争既争又抢。面对错综复杂的宏观形势，全市人民认真贯彻落实科学发展观，在省委省政府和市委市政府的坚强领导下，一心一意谋发展，同心同德促和谐，经济社会继续平稳较快发展，实现了“十二五”良好开局。

　　一是经济总量实现大跨越。生产总值连跨七、八两个百亿台阶，达850.5亿元，其中天长达185亿，定远、凤阳跨入“百亿俱乐部”。财政收入突破百亿，达125.8亿元，其中天长超20亿，凤阳、全椒、来安超10亿。13个乡镇财政收入超亿元，新增7个，其中铜城镇超4亿元。工业总产值在上年超千亿的基础上再迈大步，超过1500亿元。固定资产投资跨过600亿元，达690亿元。外贸进出口超11亿美元，社会消费品零售总额超250亿元。与上年比，生产总值稳居全省第六，财政收入和规上工业增加值稳居全省第七、第八，地方财政收入、固定资产投资分别从全省第六上升到第四、第五，直接利用外资从全省第十五上升到第五。

　　二是“速度滁州”实现大突破。生产总值增长14%，规模以上工业增加值增长24.1%，增速分别从全省第六和第八上升到第四；财政收入增长39.1%，再创新高，增速从全省第十上升到第五；固定资产投资增长32.4%，增速从全省第五上升到第四；社会消费品零售总额增长18.6%，从全省第十上升到第五；直接利用外资增长1.7倍，增速从后进变为先进并领跑全省。

　　三是四大收入实现大丰收。直观反映经济发展的财政收入、企业收入和城乡居民收入全面增长。全市财政收入年增量在连续三年10亿元级和上年20亿元级的基础上达到35亿元，相当于2006年总量。各县市区和省级以上工业园区财政收入增速普遍超过40%。年销售收入超亿元的工业企业新增140家，总数达350家，其中17家超10亿元，11家超20亿元，天大、安康双超50亿元。城镇居民人均可支配收入17918元，农民人均纯收入7017元，均增长18.6%。

　　四是创先争优实现大提升。贯彻中央“创先争优”号召和省委“走出六条新路”部署及市委“双超”要求，掀起比学赶超热潮。放眼全国，一批工作实现了创新晋级。承接产业转移示范区是国家战略，我们作为“一号工程”，取得突出成绩，全省现场会在我市召开，特别是引进苏州新加坡工业园，对承接产业转移具有里程碑意义。市经济技术开发区艰苦创业，成功晋级“国家队”。推进科技创新，年高新技术产业产值和授权专利均居全省第四位，与合芜蚌同获全国科技进步先进市殊荣。关心弱势群体，实施法律援助，司法部高度评价，并在全国推广。高度重视中小企业贷款难，创造性地开展商标质押贷款，总量超过10亿元，居全国地级市第一。作为全省驻军最多的地区，“双拥”工作走在南京军区前列，实现全国双拥模范城“四连冠”。完善基层卫生机构绩效考评机制，推进乡村卫生一体化管理，基层医改工作得到国家医改办肯定。在前三届农歌会成功举办的基础上，努力推陈出新，第四届中国农民歌会激情唱响，广获好评。顺应网络时代特点，高度重视民意，实施网络问政，政府门户网站在全国绩效评估中高居地级市第22位和安徽第一位。同步推进计划生育和人口素质提升，荣获全国婚育新风进万家活动示范市，南谯、全椒荣获全国人口计生优质服务先进县。保持对犯罪的高压态势，刑侦工作荣获全国先进，网上追逃居全省前列，市区刑事与治安案件双双下降。扫黄打非工作和全民健身工程均获评全国先进单位。对比全省，一批工作实现了创先进位。面对激烈的县域竞争，各地奋勇争先，天长、全椒分获全省科学发展先进县一类县第四名和二类县第一名，取得历史最好成绩。面对罕见的春夏连旱，我们全力抗灾，实现粮食六连丰，收购量全省第一，超过全省三分之一。中央一号文件出台后，我们抢抓机遇，大干快上水利项目，水利投资全省第一。大力发展现代农业，农民合作社数量、绿色食品品牌、拖拉机保有量、农机综合作业水平都居全省前列。我们坚持城镇化战略，市政建设投资全省第二，房地产开发主要指标均居全省第三，凤阳、定远、全椒县城总体规划获全省表彰。房屋征收是全国性难题，我们认真贯彻国务院条例，最大限度向被征户政策倾斜，完成国有土地上房屋征收面积居全省前列。我们积极利用省“861”计划政策推动重大项目建设，“861”项目完成投资总量、增速均居全省前三名。盘点全市，一批工作实现了创业争优。重视低收入群体居住困难，主动将保障房建设任务从省下达的2.1万套增加到3.6万套，一年超过前三年总和，总投资69亿元，并100%开工建设。近年来，土地管理政策越来越严，我市代表安徽接受全国建设用地增减挂钩清理、五年耕地保护和土地整治等专项检查，赢得高度肯定。农村土地流转面积三年翻一番，突破百万亩。时隔五年，凤阳德力玻璃和来安金禾化工双双在深交所首发上市。市本级和天长市相继引进风投基金，实现了零的突破。4条高速公路在建，3条高速公路即将上马，是我市高速公路项目最多、投资最大、推进最快的一年。三次产业结构进一步优化，二产比重首超50%，达到51.7%。

　　围绕又好又快发展，我们组织开展“十大战役”，在各条战线上全面发力给力：

　　（一）突出抓好难题破解，要素供给基本保障。

　　面对罕见的用钱、用地、用电、用工“四难碰头”和企业成本上升、市场预期下降的“叠加夹击”，我们千方百计寻求应对之策。

　　一是信贷支持与资本融资双拳出击，保障资金需求。在全国新增贷款规模收缩的情况下，我们一手抓信贷市场，内外合作，政企联动，银企联手，全市年末贷款余额576亿元，新增110亿元，连续三年超百亿、创新高。一手抓资本市场，面向社会找资金。引进风投基金18亿元，上市募集资金13亿元，成功发行10亿元市政融资债券，引进皖江基金建设高铁站区，社会融资总额首超百亿，达150亿元。同时，着力壮大政府融资平台，市城投公司资产超310亿元，全市融资平台资产超600亿元，共同完成市场融资近百亿元。

　　二是向内挖潜与向上争取双向联动，缓解用地矛盾。面对国家土地置换政策调整和土地利用规划修编带来的用地流量从紧与用地审批从严的新情况，一方面清理闲置土地，激活“沉量”资源；开展“退城入园”和旧城改造，盘活“存量”资源；规范土地置换，顺利通过检查，用活“流量”资源。转变用地方式，建设标准厂房，节约用地；坚持项目入园，集约用地；实行分期供给，精确用地；制定供地标准，科学用地。另一方面，坚持产业导向，抓大项目，争取点供指标；引好项目，争取追加指标；招“圈外”项目，争取“独立”指标；完善置换项目，争取“挂钩”指标。通过努力，获批新增建设用地指标41005亩，与上年持平；供应建设用地24969亩，其中出让19678亩，划拨5291亩，分别比上年增长23.4%、15.7%和52%。同时，新一轮土地利用总体规划获批，城镇工矿用地规模扩大到352平方公里，超过省下达规划指标111平方公里，其中主城区超37平方公里，为长远发展开辟了更大空间。

　　三是科学调度与城乡合作双管齐下，助力用电用工。加强电力需求侧管理，错峰调峰，科学调控全社会用电，及时限制高耗能企业用电，节约用电上亿度，工业用电增幅比上年下降2.1个百分点，实现了用电保障和能耗下降双赢目标。面对不断蔓延的“用工荒”和依然存在的“就业难”，市县乡村联动，内外招工并举，增加总量供给；校企密切合作，培育技能人才，化解结构矛盾。新增城镇就业7.8万人，转移农村劳动力7.3万人，分别增长31%和16.8%，招工6.6万人，实现了就业增加和用工保障双重目标。

　　（二）突出抓好示范建设，承接转移成果丰硕。

　　聚焦园区主战场，主攻项目招大商，开创了示范区建设新局面。

　　加强园区建设，承接能力全面增强。围绕先进制造业和现代服务业两大基地建设任务，深入开展园区建设突破年活动，一方面抓14个省级以上工业园区建设，新编扩编规划，实现“七通一平”，完成基础建设投资65亿元，新增开发面积40平方公里。市开发区城东、城北片区加快建设，市集中示范园区15平方公里核心区建设全面启动。谋划了全椒十谭工业园和明光凹土工业园，推动市开发区与来安共建工业园。另一方面抓现代服务业平台建设，科教园区、高铁站区、琅琊山旅游区建设全面展开。在两个基地建设中，坚持先行先试，集约集聚发展，引进浙江玉环汽摩配产业园、汊河宁北车辆配件园、明光义乌产业园、琅琊华融工业园等合作共建园区34个。立足快速承接，建成标准厂房84万平方米，入驻企业140个。

　　强势招大引强，承接成果全面显现。围绕拉长加粗产业链条，培育壮大新兴产业，以央企、民企等合作为抓手，主攻大企业、大项目，引资引智引研相结合，同步承接产业、资本、技术、人才转移，招商项目体量、质量、总量明显提升。全市招商引资到位资金1060亿元，增长69%。新引进亿元以上项目371个，是上年的1.7倍，其中5-10亿元43个、10-50亿元51个、50亿元以上10个。市开发区新引进项目平均单体投资4.7亿元，投资强度223万元/亩，分别是上年的2.9倍和1.5倍。

　　聚力工程建设，承接项目全面实施。围绕六大主导产业，实施“661”行动，开展重大项目推进年活动，60项重点工程进展顺利，百个亿元以上项目竣工投产。家电信息产业，总投资3.4亿欧元的博西华新基地一期土建工程基本完成，长电科技、东菱凯琴加快建设，申花坂田、康佳同创、金帅洗衣机建成投产。新能源产业，总投资55亿元的浩德太阳能、总投资30亿元的高邮湖风电场、总投资26亿元的天康锂电池项目开工建设，总投资50亿元的吉阳高效晶硅一期和招友科技、旭腾光伏、来安龙源风电一期、明光大港风电建成投产，引进大唐电力36亿元的风光互补发电项目，与国电集团达成百亿元的风电战略投资协议，我市被省政府列为全省唯一的新能源产业基地。硅玻璃产业，引进了总投资35亿元的东方光源和力诺年产30万台太阳能热水器等项目，台玻低辐射玻璃、赛弗彩晶和钢化玻璃等加快建设。盐化工产业，定远华塑盐化工一期完成投资75亿元并投料试车，金禾化工15万吨硝酸、3万吨三胺建成投产，总投资51.5亿元的泉盛化工开工建设。装备制造业，总投资20亿元的永强汽车和总投资40亿元的长丰SUV项目开工建设，引进熔盛重工重组全柴，三年计划投资92亿元。农副产品加工业，盼盼食品、申龙食品、福润肉制品相继投产，总投资9亿元的天津宝迪、总投资2.5亿美元的美国嘉吉、总投资15亿元的银鹭食品开工建设，今麦郎结算中心迁至天长。全市新建续建项目1694个，其中新开工1218个。

　　（三）突出抓好企业发展，工业经济增长有力。

　　坚持把工业化作为发展的主动力，强化帮办帮扶，加强协调支持。一是分类调度抓运行。实施“151”工程，排出“十二五”冲刺百亿的企业10家、冲刺50亿元的企业5家、冲刺10亿元的企业50家。实施“510”计划，每个县市区和工业园区抓新投产、新开工和重大技改项目各10个，对产值、利税前10名的企业重点调度。实施破四难专项行动，帮助小企业渡难关、上台阶。二是挖潜革新促提升。一手抓现有企业通过技改、企改等做大做强。全市385家工业企业开展技术改造，完成技改投资150亿元，比上年增长 48%。一手抓新项目建设。各地优先供地、帮扶帮办，新开工5000万元以上工业项目182个，155个在建工业项目建成投产。三是着眼配套强服务。全市工业发展基金大幅度增加到8亿元，完善工业项目招商政策，积极引进生产性服务业，强化工业政策与产业配套。通过努力，工业经济破难奋进：主导产业加速集聚。六大主导产业完成投资320亿元，实现产值1330亿元，分别占总量的88%和92%，其中装备制造业产值突破400亿元，家电信息业产值超300亿元，农副产品加工业产值超200亿元。支撑作用明显提升。规模以上工业企业新增152家，总数达750家，实现增加值381亿元。全市工业化率达45.5%，对经济增长的贡献率从58%提高到60%。创新能力不断增强。强化政府科技服务。开工建设中国家电研究院安徽分院，成立安徽省柔性光电工程研究中心，填补了我市家电产业升级的关键环节。市家电与绿色照明产品检测检验中心加快建设，凤阳硅玻璃产品检测检验中心建成使用，组织百名博士、百个科研院所、百所高校滁州行，150多个产学研合作项目顺利推进。推动企业自主创新。天长昭田公司成立了全市首家院士工作站，鲲鹏模具获批国家级创新型企业，扬子空调实验室晋升国家级，全市高新技术企业新增32家，总数达67家。新创2个国家驰名商标、24个省著名商标、47个市知名商标。高新技术产业产值700亿元，获批授权专利2034件。

　　（四）突出抓好城建攻坚，城乡建设成效显著。

　　坚持工业化、城镇化“双轮驱动”，坚定不移地推进城乡建设，新增城镇人口8万人，城镇化率达43.2%，城乡面貌显著变化。

　　大滁城建设“六化”推进。一是围绕优化布局推进大滁城建设。以建设区域性中心城市为目标，完善主城区规划，在老城区和市经济技术开发区之外，重点完善布局市本级、琅琊、南谯三大政务区，科教园、高铁站、琅琊山旅游区三大功能区，琅琊开发区、市集中示范园和苏滁现代产业园三大工业区。推进路网建设，新开工建设道路96公里，进一步拉开城市框架。城南核心区8.3平方公里主次干道全面贯通，连接高铁站区的中都大道与洪武路高标准建成，科教园区初步建成两条主干道，琅琊山旅游区建成览山路，琅琊新区中心片区路网初步形成。二是围绕美化环境推进大滁城建设。丰乐亭文化公园一期、清流河景观带一期、南湖三期基本建成，南湖四期开工建设，南谯路等五条主干道完成“白改黑”，对南湖三期和一批重点楼宇实施亮化。“131”组团新增绿地432万平方米，其中主城区217万平方米。三是围绕强化民生推进大滁城建设。加快老城改造，改善人居环境。攻坚克难，完成南谯路、丰乐路和扬子大市场等“两路一场”征迁；高杆灯、水泥厂、定远路、老滁州剧院等地块加快建设；二纺机地块改造基本完成。加快推进重点民生项目。开工建设保障房2万套；建成明光路跨铁路大桥，建设清流路跨铁路高架，解决东西交通被铁路分割问题；基本打通西涧路、凤凰西路、紫薇南路等断头路，改造小街巷40条；新建供水管网64公里、燃气管道142公里、110千伏及以上线路4条，新增城市公交车52辆；完成8个内涝治理项目，基本建成北撇洪沟，城市内涝明显减少。四是围绕催化新区推进大滁城建设。加强新区综合配套，第二污水处理厂、城东污水管网和供应新区的三、四水厂开工建设；城南新区市民文化广场投入使用，市规划馆、美术馆对外开放；高铁站区建成两站一广场；科教园区城市职业学院开工建设，交通驾校和农机校一期建成投入使用；琅琊山旅游区完成北大门生态停车场建设和野芳园改造，冠景度假村进展良好；琅琊新区创业服务中心等项目主体完工，世纪花园安置房全面开工。五是围绕产业化推进大滁城建设。坚持产城一体，布局二三产业，提升城市产业支撑力。“131”组团新建续建工业项目376个、三产服务项目95个，完成产业投资超300亿元。六是围绕同城化推进大滁城建设。南谯新城百万平方米商业和安置小区、公用设施加快建设。新来城和汊河新城双城同建，房屋征收和安置房建设面积创新高，滁来路来安段高标准改造。全椒城南新区、襄河景观带和太平文化街区高水准建设。“131”组团新开工项目257个、安置房400万平方米，新开工建设道路250公里，完成房屋征收253万平方米、投资210亿元，其中主城区121亿元，全面完成“五个超百”任务。

　　县乡村建设风生水起。天长市城南新区加快发展，市区框架扩大到35平方公里，荭草湖公园全市一流、全省县级领先。明光市新明城大会战，一批高水准的商住和公共设施加快建设，建成区达23平方公里。定远县围绕老城提升、新区完善，实施了35个商住和景观等项目，中等城市形象加速显现。凤阳县大力推进凤阳新城建设，新城区路网围合面积5平方公里。六个县城新建续建项目348个，完成投资208亿元，建成区扩大31平方公里。统筹推进农村新居民点扩容配套和老居民点环境整治，完成2018个永久性集中居住区建设规划，占村庄布点规划的86%。整村推进土地整治，新增耕地2万亩。结合土地村庄整治，新建续建新居民点606个，总数达919个，6.6万户农民迁入新居。新建乡镇垃圾转运站18个，65个村庄环境得到整治。

　　基础设施建设你追我赶。全社会交通建设完成投资68亿元。蚌淮高速凤阳段及其延伸段、蚌淮高速与宁洛高速连接线、徐明高速滁州段等4条高速公路开工建设，滁马、宿扬和滁淮高速前期工作取得重大突破。312省道天扬段改建工程开工，311省道定远程桥段改善工程完工，309省道明盱段和定明段加快建设。改造县乡道路253公里、农村危桥50座，新建通村公路166公里。凤阳港完成改造。京沪高铁滁州站、定远站投入运营。完成200项城乡电网建设项目，建成投运1座220千伏、5座110千伏变电站，新增110千伏及以上输变电线路280公里，完成投资10亿元。“数字滁州”建设完成投资6.7亿元，手机用户发展到299万户，数字和有线电视用户发展到30万户。新建城镇供水管道200公里、排水管道300公里。

　　（五）突出抓好现代服务，第三产业蓬勃发展。

　　不断强化生产性服务业的支撑力，注重发挥生活性服务业的贡献力，努力培育新兴服务业的后续力，第三产业“五业”高歌，“五朵金花”竞相盛开。商贸服务业增势强劲。家电下乡和以旧换新销售额累计31.8亿元。限额以上商贸企业新增144家，总数达391家，销售额占社会消费品零售总额的比重从22.4%提高到32%。重点商贸项目加快建设。中环国际广场、现代城、大雄农贸食品城、仪邦广场等进展良好，国际商城、瑶海农机大市场、盛大装饰城、天发广场等开业运营，沃尔玛、大润发等知名商业企业相继落户。现代物流业积极拓展。香江集团南京湾项目完成投资7亿元，中粮、德邦、美国基华等知名物流企业相继入滁，华塑物流加快建设。新型金融加速完善。协议引进金融机构3家，中信银行滁州分行已获批筹建。皖东农商行和全椒、明光农合行正式开业，新筹建村镇银行4家、融资性担保公司2家，新开业小额贷款公司和融资性担保公司各3家，保险机构发展到26家，银行业金融机构总数超过60家。休闲旅游加快发展。跃龙湖、栖凤湖、卧龙湖、神山等大型度假项目加快建设。五星级酒店建设取得突破性进展。碧桂园凤凰和全椒瑞祥酒店建成运营，天长新世纪酒店即将运营，凯迪豪生、雅阁和凤阳国际大酒店开工建设，喜来登酒店即将开工，君家酒店启动五星级改造，龙尾山酒店、来安碧桂园酒店新建和金地大酒店配套升级前期工作推进顺利，十大五星级酒店布局初见端倪。县县都有四星级以上酒店即将成为现实。新创建三星级以上农家乐10家。以高铁通车为契机，大力拓展旅游市场，接待国内外游客957万人次，旅游总收入63.5亿元，分别增长53%和61%。房地产业持续增长。同步推进保障房和普通商品房建设，房地产施工面积1881万平方米，其中新开工面积707万平方米，完成投资166亿元。同时，大力推进全民创业，新增私营企业3412户、个体工商户15276户，民营经济注册资本546亿元，增长38%，天长经验被全省推广。

　　（六）突出抓好城乡统筹，现代农业加快推进。

　　扎实开展城乡统筹推进年活动，推动农业现代化与工业化、城镇化同步发展。粮林水“三块”并重，农业基础进一步巩固。水利建设完成投资14.7亿元，对3座中型和255座小型水库进行除险加固，完成25座中型水库除险加固扫尾工程，开工建设洪泽湖抬高蓄水位影响处理和清流河等9个河段工程，实施“一事一议”项目3009个。滁河综合治理先期到位资金1.75亿元。开展水稻产业提升行动和小麦高产攻关竞赛，推进百亿粮仓建设，粮食总产88.1亿斤，增长2.3%。大力发展林业产业，新增苗木花卉面积1.9万亩，森工产值达到70亿元。种养加“三业”并行，农业结构进一步优化。种植业上，在稳定粮食生产的同时，着力发展经济作物。全市蔬菜面积78.5万亩，比上年增长1.7%。甜叶菊面积超10万亩，滁菊面积超万亩。养殖业上，着力发展规模养殖，比重超过70%，肉蛋奶产量49.3万吨、水产品产量29.2万吨，分别增长2.2%和4.9%。加工业上，着力培育产业化龙头企业，新增国家级龙头企业2家，121家市级以上龙头企业实现销售收入186亿元。科技、组织和规模“三化”并举，传统农业进一步转型。推广超级稻207万亩、机插秧132万亩，分别占水稻面积40%和26%；农机总动力达602万千瓦；农民合作社新增656个，总数达2011个；农村土地流转新增28万亩，总面积达102万亩。

　　（七）突出抓好公共服务，民生福祉得到改善。

　　坚持以人为本、执政为民，发展社会事业，实施民生工程，创新社会管理，公共服务“五路联动”。全市财政民生支出149.2亿元，占总支出的81.6%，其中33项省级民生工程投入38亿元，比上年增加14.5亿元。学校建设得到加强。全力保障教育投入，全市财政教育支出29.3亿元，占总支出16%。义务教育学校标准化建设和校安工程扎实推进，182所薄弱学校条件明显改善，新加固校舍42.4万平方米、重建校舍41.4万平方米。结合校安工程和优化布局，新建中小学校区189处。学前教育三年行动计划全面启动，新建和改扩建普惠性幼儿园29所。职教大市建设大力推进，中职学校在校生增加5000人。高校建设得到加强。卫生服务加速提升。着力缓解看病难、看病贵问题，累计建成乡镇卫生院94个、村卫生室1049个、社区卫生服务中心和服务站130个，基本药品零差率销售全面巩固，新农合参合率达97.4%，报销比例进一步提高，城乡医疗救助支出5880万元，农村卫生院门诊人次上升24.3%，人均看病费用比医改前降低36%。加强高水平医院建设，建成市一院和中西结合医院医技大楼，市二院新院区开工建设，县及县以上医疗机构新增建设面积11万平方米。文化事业大力建设。新建50个乡镇综合文化站、370个农家书屋、151个城乡体育健身工程，确定首批34位非物质文化遗产代表性传人，“一校两团”基本建成，各级图书馆、文化馆、博物馆、纪念馆全部免费开放，文化下基层、进社区和特色文化社区创建等活动深入开展。就业和社会保障不断强化。加强创业、再就业和农民职业技能培训，建设农民工创业园29个，发放就业扶持资金1.3亿元。在岗职工养老、失业、医疗、工伤、生育五大保险参保133万人次，增长18.3%。城镇居民基本医疗保险、被征地农民最低生活保障基本全覆盖，城乡居民养老保险参保人数达162万。城乡低保人均月补差分别提高28%和25%，最低工资和退休人员养老金标准进一步提高。农村敬老院新增床位1304张，“五保户”集中供养率达48%。创建和谐劳动关系，2000户企业签订集体合同。改造农村危房5449户。安全饮水工程惠及34万农村人口。加强市场监管，努力稳定物价，发放临时价格补贴3600万元。社会建设有力推进。深入开展安全隐患大排查、大挂牌，专项整治重点行业，安全生产事故和死亡人数分别下降6.8%和12.2%。加强人口计生投入，完善“村为主”机制，强化过程管理，县乡村计生服务网络建设全面达标，人口自然增长率4.59‰，出生人口政策符合率92.8%。实行市区动态巡逻防控，累计建成城市电子监控点1.2万个，社会治安综合治理防控体系加快完善。此外，“六五”普法全面启动，新一届村委会和乡镇政府换届选举有序开展，天长秦栏镇、市工商局等8个单位荣获全国文明村镇(单位)称号，食品药品安全、质量监督管理、民族宗教、防震减灾、国防动员、外事、侨务、统计、气象、档案、科普、供销、红十字会、地方志、残疾人、老干部、文联、妇联及工商联等工作都得到加强。

　　（八）突出抓好节能减排，生态环境治理有效。

　　大力抓好节能工作。实行节能节电双控制，分解节能任务，每月督查调度；推行项目用能评价，实行“一票否决”；对126家年耗能1000吨以上标煤企业实行重点管理，对44家年耗能3000吨以上标煤企业开展重点核查，对高耗能企业坚决采取限制措施，全面完成了节能任务。狠抓主要污染物总量减排，建设污水管网120公里，淘汰黄标车和报废车12865辆，抓好17家规模养殖场达标排放，取缔5家重金属排放和铅蓄电池企业，减排指标完成情况全面超全省平均水平。整改严重影响环境的问题，确定重点保护水源地62个，开展西涧湖及周边环境整治，完成原子能所核装置退役验收。加强生态维护，植树造林15.7万亩，森林覆盖率26%，增加近1个百分点；森林蓄积量1500万方，增长6.6%。加强水土保持管理，完成淮河河道禁止采砂工作。

　　（九）突出抓好自身建设，创新服务措施扎实。

　　坚持依法行政，提升服务能力，努力打造人民满意的政府。一是虚心接受监督。自觉接受人大的法律与工作监督和政协的民主监督，主动接受社会和网络舆论监督，认真办理人大常委会审议意见和市政协建议案，努力办理人大议案和61件人大代表建议、361件政协委员提案。二是创新体制机制。打破体制障碍，充实一线力量。抽调260多名干部集中办公，加强大滁城等七大指挥部力量；安排200多名干部充实市帮办帮扶等六大中心。加强效能建设，强化效能督查，市效能办“三办合一”模式全面复制到县市区。开展第四轮政务流程再造，在全省率先引进17个中介服务项目进中心，实行涉企证照联合年检和土地房产联合办证，136个项目签批前移，55项行政审批取消，31项收费标准降低，各项收费水平在周边地区动态最低。三是加强学习提高。结合科学大讲堂，以中央十七届六中全会、省第九次党代会、市第五次党代会精神和前沿知识为主要内容，培训公务员上万人次。四是完善施政理念。坚持民本思想，畅通沟通渠道，强化服务手段。完善政务信息公开机制，主动公开信息20万条。进一步加强市长信箱、滁州民声网、“12345”市长热线、政风行风热线等工作，积极办理热线电话11万个。落实省“五级书记带头大走访”要求，深入开展“三民”工程大走访，建立“四包三推一联系”制度，搭建社区工作平台，办实事4600多件，解决问题5600多个。建立健全信访工作责任制，化解信访积案130起。五是加强廉政建设。制定重大行政决策规范，细化自由裁量权，着力治理关键环节、重点领域。完善开评标，严查串围标，实行电子竞价，延伸标后管理，招标采购354亿元，其中市本级208亿元，工程建设项目节约率达18%。加强审计和监察监督，核减政府投资项目资金2.1亿元，查处违规资金5432万元、违纪案件456件，处理457人。强化目标绩效管理，从严治理慵懒散乱，问责72人。

　　以上成绩是省委省政府和市委坚强领导的结果，是全市上下奋力拼搏的结果。在此，我代表市人民政府，向全市人民，向人大代表、政协委员和党代会代表，向市县两级几大班子历任领导和离退休老同志，向各民主党派、工商联、无党派人士和各人民团体，向驻滁解放军指战员、武警消防官兵和政法干警，向国家和省驻滁单位，向广大来滁投资者和建设者，向所有关心支持滁州发展的海内外朋友，表示衷心的感谢和崇高的敬意！

　　在肯定成绩的同时，我们也清醒地看到存在的问题：在发展目标普遍较多超过年初计划的同时，少数指标没有实现预期目标。经济社会发展质量还不够好，体现在产业结构不够优，战略性新兴产业发育不够；工业实力不够壮，高耗能企业比重大；服务业支撑力不够强，现代服务业发育不足；农业转型升级不够快，现代农业发展水平还不够高。此外，城市综合配套不够完善，管理水平有待提高，城市建设特别是房屋征收中矛盾较多；安全生产中道路交通事故发生率较高，较大事故起数居高不下；政府系统工作还存在薄弱环节等。这些问题，我们将高度重视解决。

　　二、2012年工作安排

　　2012年的国内外经济形势更为复杂严峻。从国外看，发达经济体主权债务危机继续深化，增长动力不足，影响我市企业出口。从国内看，通胀压力仍然存在，宏观调控没有明显放松，市场竞争更加激烈，一些刺激消费政策面临调整，加上节能减排约束进一步强化，要素保障困难和环境容量制约问题突出，企业经营环境不容乐观。面对新形势，我们要认真贯彻落实中央经济工作会议、省第九次党代会和市第五次党代会精神，以科学发展为主题，以富民强市为目标，以转型升级为路径，以示范区建设为引领，以“661”工程和大滁城建设提升年、示范区建设突破年、城乡统筹推进年活动为抓手，稳中求快，进中求好，在转变发展方式上取得新进展，在深化改革开放上取得新突破，在改善民生上取得新成效，实现“千亿滁州”，为进入全省第一方阵打下坚实基础。经济社会发展的预期目标是：生产总值增长13.5%，财政收入增长18%，全社会固定资产投资增长25%以上，社会消费品零售总额增长17%，外贸进出口总额和城镇居民人均可支配收入、农民人均纯收入均增长15%，城镇登记失业率控制在4.4%以内，人口自然增长率控制在7‰以内，全面完成省下达的节能与减排任务。以上目标，在具体执行中我们将坚持自我加压、努力追赶跨越。

　　（一）着力抢抓转移机遇，实现示范建设的新成效。

　　按照省委、省政府关于示范区建设“三年见成效”的要求，全力承接产业转移，为实力滁州、崛起跨越蓄积更大能量。

　　瞄准千亿引资，着力招大引强。围绕大高新特项目，主攻国内外500强和行业领军企业，力争引进亿元以上项目500个，招商引资到位资金1300亿元，其中工业引资超过千亿，争取利用市外资金规模和增速继续位居全省前列。一是强化任务落实。完善招商任务分配办法，将招商任务按职能、按平台分解落实到各产业、各园区、各县市区和各指挥部，把各部门的招商任务调整为牵头行业招商、捆绑产业招商、提供线索招商等。在此基础上实化措施、硬化考核。二是强化落地帮办。加强信息搜集整理，全面推行规划一张图、供地一张表、谈判一把尺、产业一盘棋，完善工作指引。建立招商项目周汇报制度，加强招商调度。招商安商并重，做好企业服务。三是强化招商手段。继续推进五大合作，加强与国家和省级“五会五联”的联系，开展精准招商，注重挑商选资，完善分享办法，鼓励园区协作招商。发挥部门优势，挖掘职能招商潜力。四是加强新兴产业招商。我市产业结构还处于形成阶段，既要重视引进精密加工、数字技术、液晶显示、感知元器件等我市有一定产业基础和关联度大的高端项目，又要无中生有，积极引进新材料、生物医药、节能环保等项目，丰富产业结构，提升产业层次。

　　瞄准两年翻番，着力拓展园区。加大园区建设力度，着力打造项目快速落地的平台载体，力争各园区固定资产投资、招商引资到位资金均增长60%以上，实现两年翻番。一是全力建设苏滁现代产业园。坚持产城一体理念，围绕“滁州浦东”目标，组织强大力量，全力服务保障，加快建设4条外部对接道路，全面拉开一期12平方公里基础设施建设，同步推进园区的国际招商。二是大力建设现有工业园区。市开发区城东开发完毕，城北初具规模，新增开发面积10平方公里。全面建成市集中示范园区15平方公里核心区路网框架。12个县属省级工业园区合力完成基础设施投资60亿元以上，新增开发面积20平方公里。加强园区生产生活配套，推进产城融合发展。积极推动市科教园区、高铁站区、全椒十谭工业园、明光凹土工业园、南谯沙河工业园争创省级园区，市开发区和天长开发区继续争创省高新区，天长开发区要争创国家级开发区。三是积极谋划新的园区。着眼分类承接、集群引进，积极创办园中园、专业园、特色园，新建标准厂房80万平方米。继续推动园区合作共建，重点抓现有协议落实和规模扩大。支持南谯区在乌衣谋划新的园区、支持琅琊开发区扩建升级，推动市开发区与全椒、来安、汊河开发区共建新园区。四是狠抓园区服务保障。全面推行园区社会事务“反托管”，行政审批实现“园区的事园区办”，把优秀人才充实到园区一线，充分保障园区发展需求。

　　冲刺千亿投资，着力建设项目。努力保障土地供给，加强项目调度督查，力争新开工千万元以上项目2000个，竣工项目1000个，实现规模以上固定资产投资860亿元，争取全社会投资超千亿。推行建设用地指标竞争制度，把好项目投资强度、科技水平、能耗高低、规模大小、投入产出关，大力提高土地使用效益。苏滁现代产业园新引进项目投资强度每亩不低于400万元，市开发区、集中示范园区每亩不低于300万元，县市区的省级工业园区每亩不低于200万元。

　　抓好以上工作的同时，要大力推动对内对外开放。巩固一般贸易，拓展加工贸易，推进公路口岸建设和水陆对接、多式联运、电子通关，继续申报出口加工区。主动加强与南京、合肥在交通、科技、流通等方面的协作。

　　（二）着力培植发展后劲，实现工业经济的新扩张。

　　冲刺全省第一方阵，工业是关键。要坚持工业强市的主战略，围绕转型升级，强化要素供给，狠抓千个新项目开工、千家企业技改、百个亿元项目竣工投产，争取完成技改投资200亿元、工业投资500亿元，实现工业总产值2000亿元、工业增加值500亿元，进一步推动产业集聚、企业做大做强。家电信息产业，开工建设格兰仕、华彩电器、万爱电器、扬子配套工业园等项目，实现博西华新基地、长电科技、东菱凯琴、中家智锐、银兴VIP芯材等项目投产，争取家电产能超过3500万台。盐化工产业，开工建设华塑盐化工二期和国强化工、阳城化工，加快泉盛化工建设，继续推进金禾化工技术改造。硅玻璃产业，开工建设东方光源光纤光缆、力诺太阳能热水器、福莱特汽车玻璃等项目，争取台玻低辐射玻璃、确成白炭黑等项目建成投产。新能源产业，开工建设吉阳科技二期、明光和来安风电二期及全椒和定远风电项目，加快建设浩德太阳能和南谯风电项目，争取天康纳米锂电池、高邮湖风力发电等项目投产。装备制造业，开工建设熔盛重工新项目，争取开工风电装备制造项目，加快推进长丰扬子SUV等项目建设。农副产品加工业，开工建设今麦郎华东新基地等项目，争取银鹭、嘉吉、六个核桃、蜡笔小新、金冠、公元等项目建成投产。抓好以上工作的同时，继续实施“三上”工程，帮助企业渡难关，争取新增规模以上企业130家，总数达到880家；新增亿元以上企业50家、50亿元以上企业2家。

　　建设创新型城市，强化工业发展支撑。大力建设创新载体。围绕主导产业发展需求，支持安徽省柔性光电研究中心建设，推进彩色柔性液晶显示器工艺工程化、集成化和技术装备建设，加快建设中国家电研究院安徽分院，建成家电和绿色照明产品检测检验中心，开工建设中国质量认证中心滁州分中心、市科技创业中心三期，谋划建设机械装备研究院。全椒、琅琊要推进科技孵化器建设，定远要筹建盐化产品检测中心，来安要筹建精细化工产品检测中心，天长要筹建光伏和电线电缆产品检测中心与科技服务公共平台。积极培育创新主体。新增市级以上企业技术中心、高新技术企业各20家以上，国家和省级创新型企业2-3家；支持长电科技、扬子空调等企业筹建国家级实验室、院士和博士后工作站，争取国家、省重大科技专项，参与制定国家和行业技术标准。深入推进产学研一体。加大财政科技投入，支持新产品开发、发明专利和科技成果转化，推动安徽科技学院、滁州学院和市外高校院所在我市领办联办研发机构，与企业建立创新联盟，开展科技攻关，重点抓好100个产学研项目。切实加强人才工作。支持高校和企业引进“两院”院士、学科带头人、专利持有人和高学历技术人员，争取引进紧缺和高层次人才100名以上。实施职教大市建设五年行动计划，支持滁州职业技术学院、城市职业学院和技师学院建设，努力创建高职示范院校、公共实训基地和全国示范性实践基地，校企合作，订单培养，培训技能人才2万人。

　　（三）着力建设新型城市，实现城乡面貌的新变化。

　　把城镇化作为拉动内需的最大动力、最强支撑，顺应高铁时代到来和融入南京、合肥两大都市圈的大势，按照打造新型区域中心城市的目标，坚定不移地完善提升城乡建设。

　　深入推进大滁城建设，提升中心城市。按照“立足惠民、提升出彩、完善功能、适度拓展”的要求，“131”组团计划实施建设项目200个，新建续建道路200公里以上，新建保障房200万平方米，完成投资超200亿元，力求点上出精品，线上有提升，面上展形象。一是抓好城南核心区建设，展现集中连片的新区。进一步完善路网、精致绿化，建成龙蟠小区、国际城、天逸华府和海关、商检大楼等百幢高层。二是抓好老城区改造，展现星罗棋布的亮点。开工建设市博物馆，建成南湖四期、东阳桥和内城河闸站、清流高架和清流新桥。同时，成片与点状改造相结合，全面启动龙池街周边房屋征收、南湖四期增拆。三是抓好功能区建设，展现组合优化的空间。高铁站区新建续建道路7条11公里。科教园区进一步拓展主干道，续建滁州学院南校区、滁州职业技术学院新校区和滁州城市职业学院。琅琊山旅游区提升丰乐亭景区，开工建设览山路给排水工程。琅琊新区新建续建道路5公里、安置房34万平方米，高标准建设市民广场和如意湖公园。四是抓好生产生活配套，展现产城一体的模式。建成三、四水厂。加快建设城南新区三大城市综合体建设，推进高铁站区10万平方米安置房和城市综合体开发，完善市开发区、集中示范园区内产业和商住小区布局，加快布局琅琊新区重大商贸项目。五是抓好主副中心联动，展现同城发展的格局。按滁州形象、安徽亮点的要求，加快南谯新城建设。续建双洪公园和污水处理厂等项目，新建四、五期路网和青少年活动中心、柯湖生态公园等项目。按主城区标准加快来安、全椒县城建设。全椒县继续打造城南新区，改造提升老城区，积极推动土桥水库开发。来安新城全面发力，加快来安中学新校区、中央公园、来河综合治理等项目建设，开工建设二期路网和职业中学新校区等。汊河新城加快实施城乡一体化项目。推进各组团连接线改造和连接带开发，高标准改造扬子路，启动滁州高铁与南谯新城、全椒火车站之间连接线建设，推动滁全路、滁乌路、滁来路沿线组团开发，市县区合力推进高铁站区建设。继续开展“五城联创”，加强市容环卫治理，整治交通秩序，严控城乡违法建设。

　　继续抓好县乡村建设，提升县域水平。 一是突出功能完善，提升县城。一方面，加强老城区水电路气、公园绿地、环境卫生、文化体育等公共服务配套，稳步推进棚户区改造。另一方面，按照中等城市规模要求，着力完善提升新区。天长市要进一步拉开城南新区框架，定远县城东新区要出新形象，明光市要加快新明城建设进度，凤阳县要加快充实凤阳新城，四个县城要合力完成投资100亿元以上。二是突出集聚能力，提升乡镇。按照镇当城建的思路，继续推进30个左右经济强镇和扩权强镇试点镇建设。扶持边界乡镇、人口大镇发展商贸经济。制定山区乡镇发展规划，因地制宜发展生态经济。三是突出社区建设，提升村庄。新建续建343个新农村居民点，扩大规模，完善配套，建成100个新型社区。加强农村垃圾治理，每个县市区要新开展2个乡镇垃圾转运站试点，所有试点乡镇的新居民点要全面推行垃圾统一收集。

　　加强城乡基础设施建设，提升承载能力。发展大交通、大物流、大网络。交通建设“双百”工程力争新建续建100个项目、完成全社会交通投资100亿元。加快徐明高速滁州段、蚌淮高速凤阳延伸段和连接线建设，建成蚌淮高速凤阳段，争取开工建设滁马、宿扬高速滁州段和312省道天釜段、天长至东阳段。启动滁宁快速通道滁州段和104国道滁州至汊河段拓宽前期工作。推进京沪高铁定远站连接线建设。开展清流河航道疏浚、滁州港建设前期工作，抓好汊河闸改造、淮河凤阳鸿运港二期和凤玻公司、天长天然公司、明光光达建材三个码头建设，带动“两河”沿岸开发。实施新一轮城乡电网建设改造，扩建220千伏和110千伏变电站各2座，新建220千伏变电站1座、110千伏变电站6座，新增110千伏及以上输变电线路80公里；完成电力建设投资7亿元左右。继续推进TD-SCDMA等第三代数字通讯项目建设。

　　切实加强规划工作，提升建设品位。适应苏滁现代产业园建设需求，启动新一轮城市总体规划和土地利用规划修编。围绕建设精致大气的品质城市，加强城市基础设计和专项规划工作，重点深化对城市天际线、高层区、快速交通等专题研究。树立有机更新的理念，城乡建设注意挖掘历史文化底蕴，保护历史文化遗存。全面梳理城乡古村镇、古民居、古遗址、古树名木并制定保护措施，启动滁城东关、天长龙岗、南谯乌衣老街、全椒太平桥和袁家湾等历史文化街区规划工作，加强清流关和嘉山抗日政府旧址保护。新农村建设注意保护历史自然风貌，保留村庄记忆。

　　（四）着力拓展现代服务，实现三产结构的新优化。

　　以现代服务业为引领，大力推进服务业重点项目和集聚区建设，扩大服务业规模，优化服务业结构，提高支撑力与贡献力。

　　强力发展物流业。以建设区域性物流节点城市为目标，积极抓好物流园区规划建设、物流节点开发利用和物流企业培育引进工作。依托淮河、滁河、4条铁路和10条已建、在建、待建高速公路，规划建设滁州综合物流、天长低碳物流、凤阳港口物流、汊河商贸物流、明光工业物流、定远盐化物流六大园区，建设一批站场和仓储设施。积极引进全国知名的第三方物流企业，开展大型工业企业服务业分离试点，推动本地物流企业争创A级。

　　大力拓展金融业。以扩张社会融资总规模为目标，着力提升金融工作水平。一是巩固间接融资。强化银行与企业、银行与政府、银行与银行、银行与融资性担保机构的合作，争取新增贷款再超百亿。二是拓展直接融资。积极争取股票融资、债券发行、融资租赁、BOT融资，继续适度利用BT融资，推动发行短期融资券、中期票据、中小企业集合债和资产证券化，充分运用信贷替代产品，争取直接融资超200亿元。三是壮大融资平台。投融资并举，进一步充实市县两级融资平台资本金，做大做强市开发区、市集中示范园区、市高铁站区、琅琊山旅游区融资平台，探索建立水利建设融资平台，推动市开发区同创公司发行债券，争取全市融资平台资产规模达到800亿元。四是发展金融机构。力争新引进股份制商业银行、风险投资基金各1家，新筹建和开业村镇银行3家，全面完成县信用联社组建农商行工作，争取全市银行业金融机构发展到70家左右。积极支持保险和证券业发展。

　　着力提升商贸业。以建设大市场、大商场为目标，重点抓好世贸广场、盛辉99广场、天长财富广场、定远汽配城、来安新城、全椒江海、凤阳洛兹、明光时代广场等商业综合体，国际商城、瑶海农机大市场、大雄农贸食品城、南京湾等市场建设。着力培育限额以上商贸企业，力争新增100户。继续开展农贸市场标准化改造，滁城完成4个，每个县城启动1个。积极发展连锁、配送、冷链、“人脑+电脑”的网络销售等新业态，探索实体经济、电子商务与现代物流相融合的新模式，加快改造传统服务业。

　　聚力打造旅游业。深入开展旅游四项行动，提升旅游吸引力和供给力。新型景区开发行动，重点抓好冠景、皖东风情小镇、跃龙湖、栖凤湖、金牛湖、神山、龙山、大金山滴水寺、女山和凤阳山地质公园等项目建设。高星酒店创建行动，加快凯迪豪生、喜来登、水岸星城、雅阁、凤阳新城等项目建设，争取碧桂园凤凰、瑞祥等正式申报评定星级。传统景区提升行动，积极推动白鹭岛、韭山洞、明皇陵创建4A级景区，龙岗抗大八分校、儒林文化公园、皇甫山、九天峰创建3A级景区。休闲产品打造行动，大力推进旅游与文化、体育、教育、农业等产业融合发展，培育一批慢生活方式的休闲娱乐、特色餐饮、精品农家乐和文教体旅游项目，打响中国休闲城市品牌。

　　合力发展房地产业。继续抓好保障房建设，续建2.4万套，新开工3.6万套，其中公租房1.87万套。改造城市和工矿棚户区1.5万套、国有林场危房1130套、农村危房7000 套，积极争取列入利用公积金贷款支持保障性住房建设国家试点城市。稳步推进普通商品住房建设，大力发展商务楼宇、工业地产和旅游地产，争取房地产新开工面积超过600万平方米。

　　协力发展文化产业。围绕创意设计、数字出版、动漫游戏、演艺娱乐等，建立文化产业重点项目库，规划建设琅琊山、凤阳明文化、全椒儒林文化、天长孝文化四大基地和一批文化市场。支持市广播电视台、滁州日报社、市演艺公司做大做强，推进体育产业化，引进文化产业战略投资者。不断提升琅琊山庙会、走太平、花鼓节举办水平，对非物质文化遗产进行保护性开发，打响一批地方文化品牌。进一步擦亮农歌会这一国字号文化品牌。

　　全力推进集聚区建设。重点抓好滁城水石商圈、城南中央商务、琅琊山旅游、乌衣现代商贸服务、天长城南商贸服务、来安汊河商贸物流、全椒站南、定远鲁肃大道十里商业街、凤阳文化旅游、明光综合商贸等十大服务业集聚区，引导资源要素集中发展。着力培育商之都等30家重点服务企业。积极争取市级集聚区和重点企业纳入省扶持范围。

　　努力建设创业型城市。推广天长等地全民创业经验，初次创业、二次创业、本土创业、外来创业、返乡创业多管齐下，新建5个农民工创业园、8个再就业创业园，开展创业培训1万人，发放小额担保创业贷款1亿元。着力支持大学生创业，创办3个大学生创业园，支持年轻人网上创业、开设网店。力争新增工商户1万户、民营企业1000家，实现500家个体工商户“户改企”。加强政策扶持引导，推动本土企业做大做强。

　　同时，积极发展商务服务、社区服务和养老服务，争取引进中网科技等物联网项目、北京科维等云计算项目和软件、服务外包等项目。中介服务是我市突出的薄弱环节，要大力培育引进法律、会计、货代、造价、评估、监理等中介机构。继续促进城乡消费，发展夜市、健身娱乐、教育培训等消费热点。

　　（五）着力实施七大工程，实现现代农业的新进展。

　　坚持“三化”同步，积极推进农业区域化布局、标准化生产、规模化种养、产业化经营，加快发展现代农业。百亿粮仓工程，实施220万亩超级稻、260万亩水稻产业提升、130万亩小麦高产攻关等行动和20万亩玉米振兴计划，奋力夺取粮食“七连丰”。现代农业示范区创建工程，重点建设全椒、天长永丰稻麦、乌衣都市农业、女山湖现代渔业、来安现代禽业、凤阳小岗、定远池河7个主导产业型综合示范区和省级滁州农业产业化示范园，总面积超过100万亩。采用新品种、新材料、新设施、新技术、新机制，打造现代农业的大观园、农村经济的综合体和新型农民的大舞台。农业产业化转型倍增工程，围绕“十二五”农产品加工转型升级、产值倍增目标，着力培育龙头企业，力争新引进知名农产品加工企业10家，新增省级龙头企业超全省平均数。积极建设市县粮食产业园。水利建设工程，开工建设淮河入江水道高邮湖大堤加固、滁河防洪近期治理和池河防洪治理工程，新开工6座中型、100座小型水库除险加固项目，推进江巷、崔家湾、山许、靠山4座水库建设前期工作，完成驷马山、女山湖大型灌区和沙河集等中型灌区节水改造，引导农民“一事一议”清渠扩塘修站，建设高标准农田13万亩。继续推进江淮分水岭综合治理开发。林业“双百双十”工程，围绕五年造林百万亩、森工产值超百亿、建成十大苗木和十大森林旅游基地目标，植树造林17万亩，新增苗木花卉2万亩、森林旅游度假基地3个、森林旅游人家190个。经营机制创新工程，启动国有林场改革试点工作。新增土地流转30万亩、农民专业合作社200个，打造10大规模经营农庄。保障体系建设工程，完善基层农技推广体系，大力发展农机事业，扩大农业政策保险覆盖面，建设40个农村新网工程示范点。

　　（六）着力发展社会事业，实现民生福祉的新改善。

　　围绕群众反映迫切的民生需求和十大幸福工程，积极发展社会事业，实施33项民生工程，总投入超过50亿元，同时继续办好惠民实事，让发展成果更多体现在民生改善上。一是大力促进就业。把就业作为民生之本，培训转移农村劳动力和新增城镇就业各5万人。二是促进教育公平。推进教育均衡发展，提升75所薄弱学校，新建和改扩建29所普惠性幼儿园，建设100个校内留守儿童之家，为3万名高中阶段经济困难学生提供助学金，为7000名义务教育贫困寄宿生提供生活补助，完成校安工程三年建设任务，努力增加校车投入。三是改善城乡交通。全市新增公交车200台以上，总量超过800台，其中滁城新增60台以上；推进城乡客运一体化，整合改造“公车公营”农村客运班线，基本实现行政村全覆盖。四是提高医疗保障水平。深入推进基层医改，完善乡村卫生组织一体化管理，稳步推行县级公立医院改革试点，将城镇居民基本医疗保险和新农合人均财政补助标准提高到240元。五是抓好“菜篮子”工程。新建5000亩蔬菜供应基地，其中滁城保供基地1000亩；加强食品安全监管，推进农超、农贸对接，建设滁城公益性直销菜市场。六是加强特殊困难救助。进一步提高城乡低保月补差水平，社会就业与政府扶持就业相结合，动态消除零就业家庭，继续救助贫困重度残疾人和无劳动能力残疾人。七是整治小街巷环境。实施路面硬化、亮化、净化、绿化，改造小街巷150条，其中滁城50条。八是加强公共文化服务。加快推进市县两级规划馆、图书馆、文化馆建设，全面建成乡镇综合文化服务站，建设一批社区文化中心。深入开展文化下基层活动，鼓励民间文化团体自办文化活动。九是发展老年事业。新改扩建乡镇敬老院15个，增加床位1414张。探索建立社区日间照料中心，在一批居住小区安装老年健身器材。推进来安琵琶湖、全椒龙山老年产业基地建设。同时，进一步扩大城镇职工医疗、养老、工伤、失业、生育等保险的覆盖面，完善医疗救助和被征地农民社会保障，全面推行城乡居民养老保险，不断提高社会保障水平。

　　（七）着力加强社会建设，实现团结发展的新局面。

　　适应社会利益多元化、分配格局多样化的新形势，加大社会管理创新力度，把和谐滁州建设提高到新的水平。一是积极创新社会服务平台。全面推行社区网格化管理，组建社区社会管理办公室、公共服务站、社情民意信息站，提供行政审批、政策咨询等一站式服务，打造社区服务平台。全面建成县市区联合接访大厅和乡镇信访调处中心，打造民情民意平台。抓好市级应急指挥系统建设，打造应急管理平台，全面提高突发事件预防预警和应急响应能力。二是积极创新社会工作机制。继续开展“三民”工程大走访，推动省“五级书记带头大走访”活动深入；加强市长信箱、滁州民声网、政风行风热线建设，创新群众利益诉求办理机制。规范民生决策听证制度，完善职工工资集体协商、正常增长和支付保障制度，全面推行建筑劳务工资“一卡通”制度，积极探索物业管理有效途径，创新群众利益保障机制。实行矛盾纠纷滚动排查、综合研判、分流处理和分级化解，构建人民调解、行政调解、司法调解、社会调解、行业调解联动配合的多元调解机制。创新重要决策形成机制，建立重要决策社会稳定风险评估制度，充分考虑社会承受能力。三是全面抓好社会管理工作。深入开展平安滁州创建活动，加大科技强警投入，加快建设视频监控、联网报警和智能化卡口系统，构建数字化管控平台，基本建成社会治安防控体系。充实基层治安辅助力量，加大路面巡逻管控力度，严厉打击违法犯罪，增强群众安全感。深入开展“打四黑除四害”专项活动。实施“六五”普法，加强法律援助工作。开展安全生产基础基层年活动，落实企业主体责任，强化道路交通、建筑施工、消防、校园、非煤矿山、危险化学品等重点行业、重点领域隐患排查治理，整治“黑校车”，严厉打击违法生产经营行为。深入开展人口计生“村为主”百个示范村创建、百个后进村整治活动，加大出生人口性别比综合治理力度，提升人口计生工作水平。动态管理实有人口，推进基本公共服务向常住人口覆盖，认真解决流动人口就业、社保、医疗卫生、子女入园入学等问题，加强对农民工特别是新生代农民工的人文关怀和培训教育，提升流动人口服务水平。抓好以上工作的同时，大力推进精神文明创建工作，加强社会主义核心价值体系建设，引导群众增强道德判断力和道德荣誉感；认真做好企业统计改革、《滁州市志》编纂出版、省十三届运动会备战、国防教育和双拥、人民防空、民族宗教、外事、侨务、对台、档案、保密、关心下一代等工作；支持工会、共青团、妇联、工商联、科协、文联、红十字会工作；发展妇女儿童、老龄人、残疾人和慈善等事业，积极培育志愿者组织。

　　（八）着力抓好节能减排，实现环境保护的新成效。

　　深入开展低碳城市创建活动，抓好节能工作。全面推行用能评价制度，严控“两高一资”项目落地。大力推广余热余压利用、能量系统优化等节能技术和合同能源管理，建设一批重点节能工程。实行能源消费总量控制，加强能耗大户管理，大力推行公共节能、交通节能和建筑节能。深入推进国家级环保模范城创建活动，抓好减排工作，全面完成COD、二氧化硫、氨氮、氮氧化物减排任务。加强污水收集管网建设，进一步提高城市污水处理厂运行水平。严格执行环境影响评价制度，加强环境执法和重点流域水质监测，强化重点饮用水源保护，巩固提高西涧湖环境整治成果，推进国家级和省级生态村建设。推行秸秆综合利用，推广农村沼气4000户，发展循环经济。实行工业用水定额管理。保护基本农田。实施琅琊山山体生态修复工程。

　　三、着力建设奋发有为、人民满意的政府

　　我们清醒地认识到，公务员队伍依法办事的意识、务实操作的作风、破解难题的本领、化解矛盾的能力、服务各方的效果都有不少欠缺，必须采取有效措施，加强自身建设。

　　（一）在扩大公开上下功夫，着力建设阳光政府。

　　把依法行政作为政府工作的基本准则，严格按照法定权限和程序行使权力。自觉接受人大法律和工作监督和政协民主监督，认真贯彻市四届人大五次会议决议和市人大常委会决议、决定，支持民主党派参政议政，努力办好人大议案、代表建议和政协委员提案。主动接受舆论和群众监督，及时回应社会关切。深入推进执法公开。进一步规范自由裁量权，实行行政执法案卷评查制度，通过政府网站及时公布投诉和执法结果，让行政执法直接接受社会监督。深入推进办事公开。推行政府行政审批、招标采购、资产处置、公务员和事业单位人员招聘工作流程网上公开，逐步推行网上办事。深入推进信息公开。进一步完善机制，加大政务信息公开力度，依法应公开的规范性文件公开率达100%。创办政府微博，加强互动交流。深入推进重大决策公开。把公众参与、专家论证、风险评估、合法性审查、集体研究作为重大决策的必经程序，建立决策纠错机制，邀请第三方对决策效果实行评估。

　　（二）在学习培训上下功夫，着力建设能力政府。

　　针对公务员队伍中存在的“老办法不能用、新办法不会用”的问题，努力提升广大公务员谋划操作、破解难题能力，打造敢打硬仗、善于突破的攻坚力量。一是加强培训。结合滁州科学发展大讲堂，分系统举办专题短训班，帮助广大公务员更新知识、拓宽视野。二是加强锻炼。全面推行政府部门领导班子成员包保联系制度，政府各部门每个领导班子成员都要帮扶一个企业、联系一个社区（行政村），到一线了解情况、感受压力、寻找解决问题的有效途径，提升执行力。三是加强考评。以实绩论英雄，以省在我市开展政府绩效管理试点为契机，积极探索政府工作量化考核制度，改进绩效考评方式，提高考评效果。

　　（三）在创新机制上下功夫，着力建设高效政府。

　　把机制创新作为提升效能的重要保障和抓手。优化职能配备。根据工作需要及时调整政府部门职能。今年重点抓好园区工作机制创新、市直部门行政审批权限整合、县市区对扩权强镇试点镇下放权力工作，让园区轻装上阵，让基层放手竞争。积极稳妥推进事业单位改革。优化流程整合。推进第五轮政务流程再造，扩大并联审批，抓好中介管理，确保收费标准周边最低、审批时间周边最短、服务态度周边最好。完善跨部门联席会议制度，推行并联操作、信息共享。优化工作方式方法。设立政府工作创新奖，鼓励政府各部门创新破解难题，在全省、全国创出先进经验。

　　（四）在平台搭建上下功夫，着力建设服务政府。

　　重点建设四个直通车，提升服务效率，改善服务质量。一是建立服务群众的直通车。进一步整合资源，拓展“12345”市长热线，强化生活服务、困难救助和交通气象等信息发布功能。完善“12316”新农村服务热线，为农民提供政策、技术和信息服务。二是建立服务企业的直通车。依托中小企业服务中心和企业帮扶服务中心，成立企业融资与用工、用电、用气服务的大平台，一揽子对接企业需求。三是建立服务项目的直通车。以项目帮办服务中心为平台，建立重大项目建设“绿色通道”，完善单位领导带头领办、工程建设全程帮办、单项困难集中交办、重要问题联合会办的“四办”机制，确保项目服务质量优、效率高。四是建立服务基层的直通车。推进电子政务建设，开展行政服务标准化建设试点，取得经验后逐步推开。

　　（五）在严格管理上下功夫，着力建设清廉政府。

　　我市正在积极推进大建设、大发展，政府部门管理的项目多、资金使用数额大，要高度关注腐败行为易发多发领域，切实加强廉政管理。一是抓好招投标等关键节点管理。全面推行政府采购和工程建设预发布、广邀商、电子竞价制度，严防严处串围标。二是抓好政府投资的项目管理。强化预决算，严控费用超标。加强工程监理，邀请热心群众担任公共财政项目社会监督员，并将其意见作为验收的重要依据。三是抓好土地出让和财政专项等资金管理。完善土地依法出让制度，加大土地出让金收缴力度。进一步整合财政资金，集中力量办大事。改革财政项目资金分配方式，推行竞争性使用，加强事后评估，确保预期效果。四是抓好拆迁安置房等公共资源分配管理。完善程序，规范操作，公开分配，确保公正公平。健全矿产资源和国有资产挂牌出让、竞争招标制度，加强城市改造中的资产评估监督，防止国有资产流失。抓好以上工作的同时，切实加强节约型机关建设，严控行政经费支出。加大行政监察、财政监管和审计监督力度，对违法违纪行为严肃查处。

　　各位代表，我们既处在大有可为的战略机遇期，也处在矛盾凸显期和经济波动期，使命光荣，任务艰巨。让我们紧密团结在胡锦涛同志为总书记的党中央周围，在省委、省政府和市委的坚强领导下，锐意进取，再接再厉，趋利避害，化危为机，坚持稳中求进，实现好中求快，为冲刺全省第一方阵努力奋斗，以优异成绩向党的十八大献礼！
